# Role of Elastic Intramedullary Nails in the Stabilisation of Displaced Midshaft Clavicular Fractures in Adolescents: A Case Series

**DOI:** 10.7759/cureus.101054

**Published:** 2026-01-07

**Authors:** Taiceer Abdulwahab, Subhashree Ravi, Murad Abdunabi, Mays Alameer, Anastasios Kelekis, Hesham Alkhateeb

**Affiliations:** 1 Orthopaedic Surgery, King's College Hospital London, Dubai, ARE; 2 Trauma and Orthopaedics, Mediclinic City Hospital, Dubai, ARE; 3 Plastic Surgery, Mohammed Bin Rashid University of Medicine and Health Sciences, Dubai, ARE; 4 Orthopaedics, Mediclinic City Hospital, Dubai, ARE; 5 General Surgery, American Hospital Dubai, Dubai, ARE; 6 Radiology, Mediclinic City Hospital, Dubai, ARE; 7 Orthopaedics, Emirates Specialty Hospital, Dubai, ARE

**Keywords:** adolescents, case series, clavicle, fracture, intramedullary, nailing

## Abstract

The aim of this study is to investigate the post-operative functional outcome following the use of titanium elastic intramedullary nailing for non-comminuted, displaced midshaft clavicle fractures in adolescents with greater than 20 mm shortening. Clavicular fractures are common acute shoulder injuries. In children and adolescents, mid-shaft clavicular fractures are managed non-operatively due to the better healing tendency of the bone. However, adolescents are commonly involved in high-energy sports and are poorly compliant with non-operative measures, which could affect healing. Studies have evaluated the use of operative techniques for adolescent clavicular fractures and reported good outcomes.

A series of four adolescent patients (ages 10-18; three males and one female) was reviewed retrospectively at our private tertiary hospital. All patients had post-traumatic displaced mid-shaft clavicular fractures treated using intramedullary nailing. The follow-up period ranged from 5.5 to 12 months. The patients were assessed for clinical and radiological union, as well as shoulder functional outcomes. All four patients demonstrated radiological and clinical union at an average of 10 weeks. The average follow-up was 7.8 months. The average QuickDASH score was 1.24 at an average of 11.5 months post-initial injury.

There were no recorded complications intra- or post-operatively, apart from one patient who developed medial nail protrusion, which required medial trimming. All patients had their elastic nails removed at an average of 15 weeks. In our series, elastic stable intramedullary nailing (ESIN) is safe and minimally invasive, with excellent patient satisfaction, cosmetic appearance, and overall outcome. We recommend its application in the surgical management of adolescents presenting with displaced mid-shaft clavicle fractures in clinical practice.

## Introduction

Fractured clavicles are among the most frequent acute shoulder injuries, with the majority involving the midshaft region [1]. Traditionally, both displaced and undisplaced midshaft fractures in adults and children have been managed conservatively [2]. Absolute indications for operative fixation include open fractures, tenting of the skin, neurovascular compromise, and floating shoulder. Relative indications include more than 100% displacement or >2 cm of shortening [3]. In younger patients, particularly children, simple immobilisation methods such as a sling or figure-of-eight brace are generally effective due to the bone’s strong healing potential [2].

There is increasing evidence that surgical management of displaced midshaft fractures in adults can achieve more predictable anatomical alignment and reduce the risk of malunion [4,5]. Operative techniques, such as internal fixation with plates or intramedullary devices, have also been applied to older adolescents, with reports of improved pain relief, earlier return to daily activities, and favourable cosmetic results [4,5].

In children under 10 years of age, the thick periosteum and strong remodelling capacity typically allow even displaced fractures to heal without complication [2]. Adolescents, however, while having more active bone metabolism than adults, may show reduced compliance with conservative treatment, particularly if engaged in high-demand sporting activities [6,7]. They may also require a longer treatment period than younger children, with a corresponding risk of prolonged discomfort and suboptimal cosmetic appearance [6,7]. In adults, dissatisfaction with conservative management is well recognised, and a similar pattern can occur in older children or adolescents [6,7]. Current consensus leans toward nonoperative management as first-line, with operative intervention reserved for specific indications (e.g., open fractures, skin tenting, neurovascular injury, or patient-/sport-specific demands).

The present study aims to assess the efficacy of elastic intramedullary nailing for stabilisation of displaced midshaft clavicular fractures in adolescents and to evaluate its impact on pain, function, displacement, and shortening in comparison with established treatment approaches.

This article was previously presented as a meeting abstract at the American College of Surgeons Scientific Forum, San Diego, USA, in October 2022.

## Case presentation

Four adolescent patients (three males and one female) between the ages of 10 and 18 years underwent flexible intramedullary nailing for simple, isolated, closed midshaft fractures with displacement equal to or greater than 2 cm on clavicle X-ray. All were otherwise healthy, with a mean body mass index of 18.5.

Three of the patients were right-hand dominant and one left-hand dominant; in every case, the injury involved the dominant upper limb. The mechanism of injury was a fall onto an outstretched hand during sporting activity. All were closed midshaft injuries, with intact overlying skin, no tenting, and preserved distal neurovascular function.

Procedures were carried out within 48 hours of injury by the same consultant orthopaedic surgeon, assisted by another orthopaedic colleague. A medial entry point was created through a 2 cm vertical mini-incision, with minimal disruption to the surrounding soft tissues. The elastic nails were advanced across the fracture site under image guidance, in accordance with the principles of relative stability, employing a three-point fixation method. No patient required an open reduction for nail passage (Table 1).

**Table 1:** Summary of data showing patient demographics, injuries and surgery details RHD, Right-Hand Dominant; LHD, Left-Hand Dominant; DASH, Disabilities of the Arm, Shoulder, and Hand

Patient sex	Age at time of injury	Activity causing injury	Dominance (right- or left-handed)	Duration from injury to surgery (days)	Hospital stay (days)	Duration of implant in situ (weeks)	Duration of follow-up post-removal (months)	Complications	DASH score at final follow-up (mean 7.8 months post removal, 11.5 months post injury)
Patient 1 (F)	17.4	Volleyball	RHD	4	1	19	12 months (17 months post injury)	None	3.33
Patient 2 (M)	16.4	Kick-boxing	LHD	1	1	13	6 months (9 months post injury)	None	0.00
Patient 3 (M)	17.8	Equestrian	RHD	4	4	13	5.5 months (8.5 months post injury)	Protruded medial nail end, trimmed on April 15, 2018	0.833
Patient 4 (M)	16.5	Cricket	RHD	2	1	15	12 months (15 months post injury)	None	0.8
Mean	17.0	-	-	3.0	2	15 weeks	7.8 months (11.5 months post injury	-	1.24

Post-operatively, patients were immobilised in an arm sling for three weeks for comfort and encouraged to perform gentle pendulum exercises. After sling removal, gradual, self-directed mobilisation of the shoulder was initiated, permitting routine daily tasks but excluding all sporting activities for an additional three weeks. Supervised physiotherapy supported this phase. Contact and non-contact sports were restricted for a total of eight weeks.

Implants were removed at a mean of 15 weeks (range: 13-19 weeks). In one case, implant removal was delayed due to the patient's travel. One patient developed medial nail tip prominence, which required trimming.

Following removal, each patient underwent clinical assessment of wound healing, neurovascular integrity, and function, with Disabilities of the Arm, Shoulder, and Hand (DASH) scoring performed by the second author at a mean of 7.8 months post-removal (range: 5.5-12 months post-removal). The DASH score was selected as the primary outcome measure due to its broad clinical acceptance and validation [8]. No cases demonstrated sensory loss over the incision site or chest wall.

**Figure 1:**
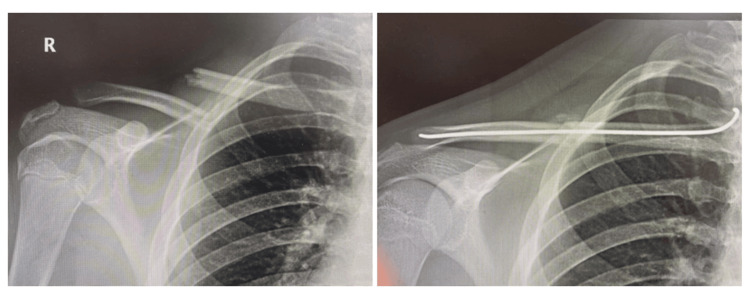
Comparison of X-rays between the initial clavicle fracture (left) and the post-operative placement of the intramedullary nail, showing 100% radiological union (right) Radiograph clavicle of Patient 1

Radiographic review at a mean of 11.5 months confirmed fracture union with callus formation in all patients, as verified by an independent radiologist (Figure 1). Scar appearance was assessed by an independent plastic surgeon, who reported excellent healing in every case.

## Discussion

In this series, four adolescent patients underwent intramedullary nailing for isolated, displaced midshaft fractures of the clavicle. Both subjective feedback and objective assessments demonstrated excellent functional recovery. Internal stabilisation allowed restoration of clavicle length, with an anticipated earlier, gradual return to activity in comparison to conservative management, although a comparison conservative management group would be required to confirm this. All individuals resumed daily activities without pain and returned to sporting pursuits, including volleyball and swimming, within a short timeframe.

Existing literature highlights the benefits of intramedullary fixation in older children and adolescents with displaced fractures of the clavicle [9-12]. One prospective study advised its use in those above the age of 10 years [7,9,10], noting advantages such as reduced discomfort, faster rehabilitation, earlier mobility, and enhanced shoulder range. For skeletally immature patients, fixation techniques designed to safeguard the growth plate are recommended [1], with the placement of elastic stable intramedullary nails (ESIN), spanning the metaphyseal ends, described as one such method [13]. Although universally accepted surgical criteria are lacking, commonly suggested indications include marked displacement, considerable shortening, or skin compromise [14]. Technical guidance also includes limiting visible nail protrusion to no more than 5 mm and restricting sporting activity for up to eight weeks [6].

Complications reported in association with intramedullary nails include bending or breakage of the implant, particularly in cases where patients returned to contact sports prematurely [15]. This underlies the standard recommendation to avoid general sporting activity for at least four weeks, and contact sports for approximately eight weeks [15], a protocol mirrored in the current study. A review of seven adolescent patients managed with this technique for acute, displaced, non-comminuted midshaft fractures documented two cases where open reduction was required to facilitate nail passage, and one case of skin irritation over the entry site, despite the use of an end cap, which led to early removal in the clinic [4,6].

More severe complications have also been documented. Luo et al. described the case of a 16-year-old with polytrauma following a road traffic accident, in whom nail breakage resulted in non-union, confirmed radiographically seven months after surgery [16]. Revision with plate fixation and autologous bone grafting was required to achieve union [16]. No such complications occurred in our cohort, though this report illustrates a recognised limitation of the method.

The present series has some limitations. The small patient group restricts the applicability of the findings and limits variability in the sample. Being retrospective in nature, the study lacks the strength of higher-level evidence. Moreover, the relatively short follow-up period may not capture late changes in function. Larger, prospective, multi-centre, randomised controlled trials are needed to define clear, evidence-based guidelines for surgical fixation in adolescents and children with displaced clavicle fractures.

## Conclusions

Based on the findings of this series and the supporting evidence in the literature, the authors advocate intramedullary nailing as the preferred operative option for non-comminuted, displaced midshaft clavicle fractures in adolescents demonstrating more than 20 mm of shortening or displacement. This technique offers stable fixation through a minimally invasive approach, with a low complication profile when performed with appropriate surgical expertise. Post-operative management should incorporate a structured and progressive rehabilitation programme, supervised by a musculoskeletal physiotherapist, to optimise functional recovery and restore a full range of motion.

In our experience, this approach provides promising preliminary results, enabling a safe and predictable return to normal activities, including competitive sport, within a shortened recovery timeframe compared with traditional non-operative methods. This is in keeping with previously referenced comparative studies. Additionally, the limited soft tissue disruption associated with intramedullary fixation yields cosmetically favourable scarring, which is an important consideration for adolescent patients. Given its reliability, patient satisfaction, and favourable functional and aesthetic outcomes, intramedullary nailing represents a compelling treatment strategy for appropriately selected cases.
